# Cardiac Startle Response and Clinical Outcomes in Preschool Children With Fragile X Syndrome and Autism Spectrum Disorder

**DOI:** 10.3389/fpsyt.2021.729127

**Published:** 2022-01-03

**Authors:** Jordan Ezell, Abigail Hogan, Elizabeth A. Will, Kayla Smith, Jane Roberts

**Affiliations:** ^1^Department of Psychiatry and Behavioral Sciences, Medical University of South Carolina, Charleston, SC, United States; ^2^Department of Psychology, University of South Carolina, Columbia, SC, United States

**Keywords:** pre-school, intellectual disabilities (ID), anxiety, heart activity, physiological startle, autism spectral disorder (ASD), fragile X syndrome

## Abstract

**Objective:** Poor physiological regulation in response to threat is linked to multiple negative developmental outcomes including anxiety, which is highly prevalent and impairing in young children with neurodevelopmental disabilities like fragile X syndrome (FXS) and autism spectrum disorder (ASD). The present study contrasted cardiac startle response in pre-school-aged children with FXS, with and without ASD, to children with non-syndromic ASD (nsASD) and neurotypical controls (NT). The relationship of cardiac startle to non-verbal mental age (NVMA), ASD severity, and parent-reported anxiety was also examined.

**Method:** Four age-matched groups of pre-school children participated including those with FXS without ASD (FXS-Only, *n* = 21), FXS with ASD (FXS+ASD, *n* = 17), nsASD (*n* = 42), and NT children (*n* = 27). Participants viewed a silent movie during which a single 200 ms 98-decibel white noise burst occurred. Cardiac activity was analyzed for pre-stimulus respiratory sinus arrhythmia (RSA) and the inter-beat intervals (IBI) at the auditory stimulus and 10 s post-stimulus. The Spence Pre-school Anxiety Scale, Autism Diagnostic Observation Schedule-2nd Edition, and Mullen Scales of Early Learning were examined in relation to startle response.

**Results:** The nsASD group demonstrated heightened cardiac activity at the auditory stimulus and 10 s post-stimulus compared to the NT controls. Neither of the FXS groups showed differences from any other group. Higher pre-stimulus RSA was associated with reduced cardiac response across groups, while the relationship between cognitive ability and ASD severity to cardiac response varied between groups. Parent-reported anxiety was not associated with cardiac response for any group.

**Conclusion:** These findings demonstrate group distinctions in cardiac responses to auditory startle. Although FXS and ASD share behavioral characteristics, the nsASD group showed a heightened cardiac startle response compared to the NT group that was not present in the FXS groups with or without ASD. Non-verbal mental age was associated with greater stimulus or post-stimulus reactivity for all groups except the FXS+ASD group, which showed no association between startle response and any clinical outcomes. Increased understanding of the relationship between physiological regulation and clinical outcomes will assist in identifying the timing and targets for effective interventions for individuals with neurodevelopmental disabilities.

## Introduction

Physiological regulation during threat is a critical adaptive response formed early in development. When a threat is experienced, the autonomic nervous system (ANS) activates the sympathetic branch in response, while the parasympathetic branch supports the body in recovering to a baseline state after the threat has passed. Well-integrated physiological regulation is related to a range of positive outcomes including better language skills, increased social responsiveness, peer engagement, emotion recognition, healthy social attachment, and social approach (1, 2). In contrast, physiological dysregulation is linked to a litany of maladaptive outcomes including emotion dysregulation, social deficits, delayed adaptive skills, and a range of psychological disorders (3, 4). Specifically, a heightened physiological response and reduced modulation in response to threat, like an auditory startle, can be indicative of anxiety (5, 6). Individuals with neurodevelopmental disabilities are at an elevated risk for physiological dysregulation and emotional difficulties, despite differing etiologies. Because there is a clear relationship between physiological regulation and developmental outcomes (7, 8), studying this phenomenon in individuals with neurodevelopmental disabilities can provide unique insights into the biological mechanisms of anxiety.

One way to capture physiological regulation is to measure heart activity in response to threat. Two cardiac indices of physiological regulation are inter-beat interval (IBI), defined as the time between heartbeats and an indicator of heart rate, and respiratory sinus arrhythmia (RSA), the temporal variation in IBI synced with respiration. When faced with an unpredictable or sudden threat, a specific pattern of physiological reactivity, known as the startle response, is often observed. This startle response is considered a primitive, elicited response to intense or sudden stimuli that prepares the body for “fight or flight” (9). During a startle response, the sympathetic nervous system is activated, allowing blood flow to move more rapidly to the extremities and breathing to increase, which allows the body to mobilize a response. During this phase, both reduced IBI (i.e., less time between heartbeats), and reduced RSA (i.e., less variability in the time between heartbeats) are typically observed. After the threat has passed, the parasympathetic nervous system becomes activated, which causes breathing to slow and blood flow to return to central organs (10). Additionally, IBI and RSA both increase as they return to baseline “resting state” levels.

Individuals with anxiety often show an atypical physiological startle response. For example, adults and children with anxiety exhibit lower resting RSA, which leads to the sympathetic system over-responding to threat, causing an exaggerated startle reflex of increased muscle tension, blink response, RSA withdrawal, and galvanic skin response. Individuals with lower resting RSA also show a slower return to baseline state after a threat (5, 6, 11). Additionally, children with anxiety often exhibit a shorter IBI at resting state and a slower IBI recovery than non-anxious peers, suggesting that they have restricted autonomic flexibility in response to threatening stimuli (4, 8, 12). Evidence also suggests that lower RSA during a resting state is predictive of a heightened startle response in typical adults (11). Taken together, these studies provide compelling evidence that physiological dysregulation, particularly in response to threat, may underlie vulnerability to anxiety in neurotypical individuals. However, little work has examined IBI and RSA during a startle paradigm in children with neurodevelopmental disorders, such as autism spectrum disorder (ASD) or fragile X syndrome (FXS).

Children with neurodevelopmental disorders appear to be at elevated risk for both physiological dysregulation and anxiety. Autism spectrum disorder (ASD) is a neurodevelopmental disorder characterized by deficits in social communication skills and the presence of restricted, repetitive behaviors (13). Current estimates suggest that 1 in 54 children have an ASD, and ~40–50% will develop a co-occurring anxiety disorder (14–16). Fragile X Syndrome (FXS) is a monogenic disorder characterized by atypical social and communication skills, repetitive behaviors, and intellectual disability. Approximately 1 in 4,000 males and 1 in 8,000 females have the full mutation of FXS, which results from a mutation on the *FMR1* gene of >200 CGG repeats (17, 18). Similar to children with ASD, children with FXS are also at a heightened risk for developing comorbid anxiety disorders (50–86%) (19, 20). Children with FXS also exhibit a behavioral phenotype that is strikingly similar to ASD, with ~60% of children with FXS also meeting diagnostic criteria for ASD (21, 22). For instance, repetitive speech and behaviors, social avoidance, aberrant eye contact, and physiological dysregulation are features common to both ASD and FXS. Given the similar behavioral phenotypes but divergent etiology between non-syndromic ASD (nsASD) and FXS, cross-population studies can provide insight into genetic contributions to physiological and emotional dysregulation (2).

Studies of children with ASD suggest that autonomic dysregulation is common in this population with findings showing a persistent state of hyperarousal, though the pattern of findings are not consistent. For example, at baseline, most studies have found that individuals with ASD are hyper-aroused, showing lower RSA and higher heart rate than peers, yet some studies found no differences (2, 23). Physiological responses to threat also appear to be atypical but varied in ASD, as evidenced by hyperarousal during cognitive tasks (elevated heart rate, lower RSA), no differences in heart rate during social interactions, and a blunted heart rate during social performance tasks (lower during stress) (24–26).

Some evidence suggests that features comorbid with ASD can also influence physiological response to threat, including anxiety and intellectual disability (ID). The role of anxiety in physiological responses to stress within ASD is limited and not well-understood. In studies of children and adolescents with ASD and comorbid anxiety, a blunted physiological response to threat (i.e., lower heart rate, less electrodermal activity) has been found compared to individuals with ASD only and typically developing peers (25, 27). In addition to anxiety, differences are also evident in individuals with ASD and ID in that some studies indicate that these individuals are hyper-aroused and show little variation in HR in response to stimuli (23). Combined, these findings suggest that the coordination of the sympathetic and parasympathetic nervous system's threat response is disrupted in some way, particularly in those children who also have anxiety or ID alongside ASD. Understanding the characteristics and outcomes of children with poor physiological regulation is important because reduced resting RSA has been associated with poorer language skills and social responsiveness in children with ASD. Overall, evidence suggests that physiological regulation has critical implications for downstream social-communicative functioning (3, 23, 28).

Evidence for physiological dysregulation is generally more consistent in children with FXS than in children with ASD. Overall, individuals with FXS show a developmental effect that becomes more pronounced with age, from hypo-arousal in the first 2 years of life toward hyperarousal thereafter, exhibiting reduced IBI and RSA during resting state (2, 29). In response to threat, one study found that infants with FXS showed reduced RSA during a stranger approach paradigm, a task designed to elicit social fear where an examiner dresses in a disguise and approaches the child (30). Another study found no differences in change in IBI in response to an auditory startle between boys with FXS and neurotypical boys, but did find that older boys with FXS showed a stronger startle response than younger boys with FXS (31). Similarly, in another study examining heart activity during rest and stress, adolescents with FXS remained in an aroused state throughout rest and stress periods (i.e., reduced RSA, shorter IBI) compared to typically developing peers (32). Thus, it appears that children with FXS exhibit chronic hyperarousal through baseline arousal as well as response to cognitive or social threats, which becomes more pronounced over age. However, the link between the physiological startle response and anxiety has not been investigated in FXS. Further, information regarding the potential impacts of co-occurring ASD on physiological dysregulation in FXS is limited, although evidence suggests that individuals with ASD+FXS often have behavioral differences and decreased cognitive and adaptive functioning (21, 33). Given the differences in developmental profiles, a direct comparison of startle response between children with FXS with and without ASD could provide insight into underlying physiological differences.

Although a link between physiological response to threat and anxiety has been established in neurotypical populations, it remains understudied in clinical groups at elevated risk for anxiety, such as ASD and FXS. A clearer understanding of the cardiac startle response in individuals with neurodevelopmental disabilities and how it relates to clinical features can provide insight into the mechanistic underpinnings of negative behavioral outcomes and guide the development of targeted prevention and intervention programs. The present study is the first to assess the cardiac startle response in pre-school-aged children with nsASD, FXS with comorbid ASD (FXS+ASD), and FXS only (FXS-Only) compared to neurotypical peers (NT). Our specific research questions are as follows:

1. a Are there differences in cardiac response (IBI) to threat during an auditory startle paradigm between pre-school children with nsASD, FXS+ASD, and FXS-Only compared to NT controls? 1b. Does pre-startle RSA predict startle IBI differentially across groups?
2. Does cardiac response to startle (IBI) relate to non-verbal mental age, ASD severity, or parent-reported anxiety, and do these relationships differ by group?

Given the current evidence for cardiac dysregulation in both FXS and ASD, it is anticipated that the clinical groups will demonstrate an exaggerated physiological response to threat (shorter IBI) after an unexpected auditory stimulus compared to the neurotypical (NT) controls. Additionally, it is expected that higher baseline RSA will be associated with longer IBI during the auditory stimulus, regardless of group. Exaggerated startle is expected to be related to clinical outcomes including low non-verbal mental age, high ASD severity, and high anxiety symptoms, given the connection between poor regulation and negative outcomes.

## Methods

### Participants

Participants for this study were drawn from an ongoing NIMH study (1R01MH107573-01A1; PI: Roberts) that is focused on the emergence of anxiety symptoms in young children with neurodevelopmental disabilities. The present sample consisted of 107 children between 36 and 72 months of age divided into four diagnostic groups: non-syndromic ASD (nsASD; *n* = 42, 36 males), FXS with comorbid ASD ruled out (FXS-Only; *n* = 21, 11 males), FXS with comorbid ASD (FXS+ASD; *n* = 17, 13 males), and neurotypical controls (NT; *n* = 27, 20 males). Participants were excluded if they were born premature (gestational age <37 weeks) or had a history of seizures. Participants with FXS were confirmed to have the full mutation (>200 CGG repeats) of the *FMR1* gene through genetic records. The nsASD group had no known genetic disorders. Autism diagnoses for the nsASD and FXS+ASD groups were confirmed through a Clinical Best Estimate (CBE) review process (22). The NT sample had no known diagnoses nor family history of ASD. The FXS-Only and NT samples were confirmed to not have ASD through the CBE process. Both males and females were included in the study in order to reflect the heterogeneity of the populations.

### Measures

#### Auditory Startle Paradigm

The Anxiety Dimensional Observation Schedule (Anx-DOS) (34) is an observational measure that consists of a variety of tasks designed to elicit anxious and fearful behaviors in pre-school-aged children. During the auditory startle task, each participant watched a two-and-a-half-minute silent children's movie while wearing a heart rate monitor. Approximately halfway through the movie, a 200 ms white noise burst occurs at ~98 decibels.

#### Physiological Regulation

Heart activity data was recorded continuously through two electrodes placed onto the child's chest using the Actiwave Cardio Monitor (CamNtech Ltd., Cambridge, UK) at 1,024 Hz. To ensure uniformity among participants, a trained research assistant identified the heart activity data period of interest as 30 s prior to the auditory stimulus through 90 s after. The cropped heart activity data was visually inspected and edited off-line for artifacts, arrhythmias, and false heart periods by trained research assistants using CardioEdit software (Brain-Body Center, University of Illinois at Chicago). Mean values for RSA and IBI were extracted using CardioBatch software (Brain-Body Center, University of Illinois at Chicago). To calculate RSA values, CardioBatch samples sequential heart periods at 250 ms epochs and then de-trends the data with a 21-point moving polynomial algorithm (35). The data was then bandpassed filtered to extract variance associated with spontaneous breathing parameters (0.24–1.04 Hz). The variance was then changed to its natural logarithm to provide an estimate of RSA.

**Figure 1:**
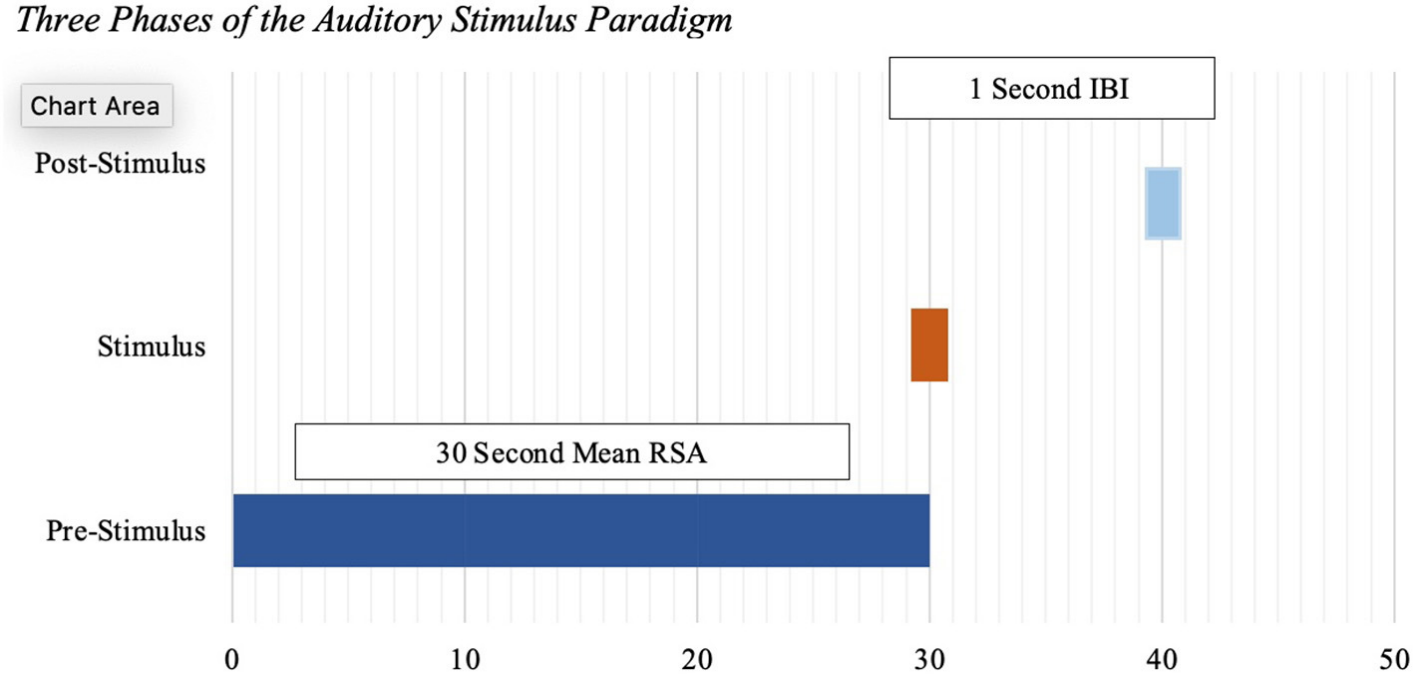
Three phases of the auditory stimulus paradigm.

Pre-Stimulus RSA was defined as the mean RSA for the 30 s prior to the auditory stimulus as a measure of baseline RSA. Stimulus IBI was the mean IBI extracted from the 1-s interval that began at the onset of the auditory stimulus. Post-stimulus IBI was the mean IBI extracted from the 1-s interval at 10 s post-stimulus (Figure 1). Pre-stimulus RSA assessed the capacity for regulation during the startle, while stimulus and post-stimulus IBI captured the acute cardiac startle response.

#### Autism Diagnosis and Severity

The *Autism Diagnostic Observation Schedule- 2nd Edition* (ADOS-2) is a semi-structured, play-based observational measure assessing the presence of autism symptomology (36). The tasks in the ADOS-2 are designed to elicit social-communication skills, and the presence of restricted, repetitive behaviors. The ADOS-2 has four modules (1–4), ranging from non-verbal to fluent verbal abilities. The ADOS-2 was administered and scored by research reliable, graduate-level professionals and reviewed by a licensed psychologist through the CBE process to confirm an ASD diagnosis in the ASD group. Site reliability was conducted on 20% of the ADOS-2 administrations (item-level inter-rater agreement = 83.3%). The Calibrated Comparison Score (CSS) is an overall severity composite relative to children of similar language abilities, ranging from 1 to 10. The CSS was used as a continuous variable of ASD severity across groups for analysis in the present study.

#### Anxiety Symptoms

The *Spence Pre-school Anxiety Scale* (PAS) (37) is a 34-item caregiver report of anxiety symptoms in children aged 2.5–6.5 years. Item scores range from 0 to 4 and summary scores are computed for Generalized Anxiety, Social Anxiety, Obsessive-Compulsive disorder, Physical Injury Fears, Separation Anxiety, and Total Anxiety. For this study, the Total Anxiety raw score was used as a measure of overall anxiety symptoms. While the PAS was developed for typically developing children, studies suggest that it is an appropriate tool for parent-reported anxiety for children with ASD because many questions target observable behaviors (38–40).

#### Developmental Level

The *Mullen Scales of Early Learning* (MSEL) (41) is a standardized measure designed to assess development from birth to 68 months across gross and fine motor skills, receptive and expressive language, and visual reception. Children with ASD and FXS often have language delays that can overshadow cognitive skills, particularly before age 5 (42). Evidence suggests that non-verbal IQ is a more stable and accurate representation of cognitive ability in young children with ASD (43). Thus, non-verbal mental age (NVMA) was used as an index of non-verbal cognitive ability for the present study. NVMA was computed by averaging the visual reception age equivalent and the fine motor age equivalent ($\frac{VR\;age\;+FM\;age}{2}$) (44). The MSEL shows good internal consistency for each subscale (0.75–0.08), and test-retest reliability (0.70–0.80).

### Analytic Plan

Statistical analyses were conducted through three phases: preliminary analysis, stimulus analysis (RQ1), and correlates of startle response (RQ2). First, in the preliminary analysis, groups were compared through one-way analyses of variance (ANOVAs) to assess for group differences in chronological age, NVMA, ADOS-2 CSS, and PAS Total Anxiety scores. Pre-stimulus IBI was also compared through a one-way ANOVA to assess for group differences in IBI prior to the auditory stimulus (i.e., baseline IBI). Second, to evaluate the cardiac response to the auditory stimulus across a 90-s task (RQ1a), multilevel regression models were run to assess group differences in IBI during the 1-s period that began at the onset of the auditory stimulus and the 1-s period occurring 10 s post-stimulus to test time-by-group interactions. The interaction was probed by centering time at the stimulus and at 10 s post-stimulus to determine any points of significant divergence in IBI trajectories (45). Because IBI increases during the pre-school developmental period, groups were matched on chronological age. Then, a multiple regression model was run to assess if pre-startle RSA predicted startle IBI with group as a covariate (RQ1b). Finally, a within-group bivariate correlations were used to determine patterns of association between PAS Total Anxiety scores, ADOS-2 CSS, or NVMA and IBI at the stimulus and post-stimulus across each group (RQ2).

## Results

### Preliminary Analysis: Group Comparisons for Age, ADOS-2 CSS, NVMA, Spence Anxiety, and Pre-stimulus IBI

Results of the first one-way ANOVA showed no significant group differences for chronological age [*F*_(3, 106)_ = 0.14; *p* = 0.94]. As anticipated, significant differences were found for ADOS-2 CSS between groups [*F*_(3, 92)_ = 78.8; *p* < 0.001], in that the nsASD and FXS+ASD groups showed significantly higher severity scores than the FXS-Only and NT groups, and the FXS-Only group showed higher severity scores than the NT group. Results of the one-way ANOVA for NVMA also showed significant differences between groups [*F*_(3, 102)_ = 24.3; *p* < 0.001]. As expected, the NT group demonstrated significantly higher NVMA than the FXS + ASD, ASD, and FXS-Only groups, and the FXS+ASD group showed significantly lower NVMA than all groups. The nsASD and FXS-Only groups were not significantly different on NVMA. For the PAS Total Anxiety Raw score, no significant group differences were observed [*F*_(3, 92)_ = 1.48, *p* = 0.23]. Lastly, results of the one-way ANOVA indicated that groups did not differ on pre-stimulus IBI [*F*_(3, 102)_ = 1.25, *p* = 0.29]. Table 1 depicts means and standard deviations for each group.

**Table 1:** Descriptive data by group.

	**Gender** **M:F**	**Age in months** **Mean** **(*****SD*****)**	**ADOS-2 CSS** **Mean** **(*****SD*****)**	**NVMA** **Mean** **(*****SD*****)**	**Spence raw total** **Mean (*SD*)**	**Pre-stimulus IBI** **Mean (*SD*)**	**Pre-stimulus RSA** **Mean (*SD*)**
nsASD (*n* = 42)	36:6	46.08 (8.0)	7.09 (1.4)_a_ (*n* = 35)	30.24 (11.2)_b_ (*n* = 40)	12.17 (10.0) (*n* = 35)	574.06 (73.48)	5.90 (1.44)
FXS-only (*n* = 21)	11:10	47.46 (7.7)	3.56 (1.8)_b_ (*n* = 18)	35.08 (10.3)_b_ (*n* = 20)	14.44 (11.1) (*n* = 18)	577.18 (57.56)	6.08 (1.25)
FXS + ASD (*n* = 17)	13:4	46.10 (9.3)	7.33 (1.5)_a_ (*n* = 15)	22.50 (5.9)_c_ (*n* = 16)	15.47 (9.3) (*n* = 15)	583.71 (105.29)	5.20 (1.99)
NT (*n* = 27)	20:7	46.55 (9.2)	2.0 (1.2)_c_ (*n* = 25)	47.59 (10.7)_a_ (*n* = 27)	9.8 (6.2) (*n* = 25)	584.89 (76.40)	6.30 (1.39)

*Group differences (p < 0.05) are indicated by different subscripts*.

### Startle Analysis (RQ 1a): Group Comparisons for IBI at Stimulus and Post-stimulus

**Figure 2:**
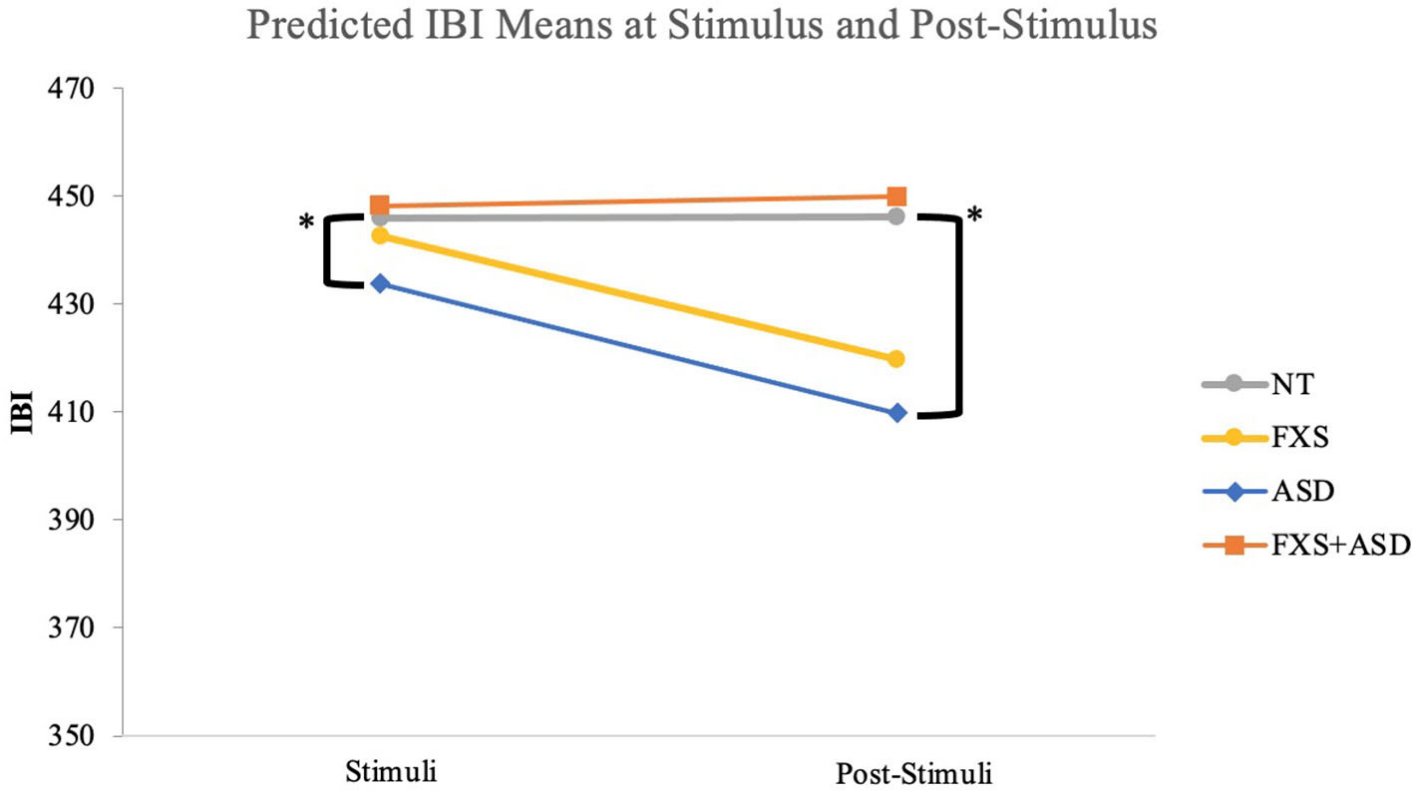
Epoch by group comparison of inter-beat intervals during startle paradigm. *Significant differences were seen between the ASD and the NT groups for stimulus (*p* = 0.03) and post-stimulus IBI (*p* = 0.02).

Multilevel regression model results with time centered at the stimulus (30 s) indicated that the nsASD group exhibited a significantly shorter IBI than the NT group (*b* = −33.856, *p* = 0.027). Neither the FXS-Only group nor the FXS+ASD groups showed significant trajectories from the NT group with time centered at the stimulus (see Table 2A; represented in Figure 2). Results from probing group-by-time interactions at 10 s post-stimulus (40 s) indicated that the nsASD group continued to display a shorter IBI than the NT group (*b* = −35.120, *p* = 0.026). Further, the FXS-Only group and the FXS+ASD groups continued to show similar IBIs to the NT at 10 s post-stimulus. The interaction of IBI-by-time was not significant indicating that this group difference was present regardless of time (see Table 2B). The reference group was recoded in subsequent models to yield estimates for differences between the clinical groups in cardiac reactivity. These results indicated no significant differences between the three clinical groups for IBI at the stimulus or post-stimulus (*ps* > 0.594).

**Table 2A:** Regression model centered at stimulus.

	***b***	**SE(*b*)**	***P***
Intercept	609.445	13.190	<0.001
Epoch 30 s	0.014	0.014	0.90
FXS + ASD	−25.850	21.22	0.23
FXS-only	−27.508	19.94	0.17
nsASD	−35.501	16.91	0.038
FXS + ASD x Epoch 30 s	−0.003	0.17	0.99
FXS-only x Epoch 30 s	0.060	0.16	0.71
nsASD x Epoch 30 s	−0.126	0.14	0.36

**Table 2B:** Regression model centered at post-stimulus regression.

	***b***	**SE(*b*)**	***P***
Intercept	609.59	13.25	<0.001
Epoch 40 s	0.014	0.11	0.90
FXS + ASD	−25.88	21.31	0.23
FXS-only	−26.91	20.03	0.18
nsASD	−36.76	16.98	0.032
FXS + ASD x Epoch 40 s	−0.003	0.17	0.99
FXS-only x Epoch 40 s	0.060	0.16	0.71
nsASD x Epoch 40 s	−0.126	0.14	0.36

### Relationship Between Pre-stimulus RSA and Stimulus IBI (RQ 1b)

Results from the multiple regression model assessing differential associations of pre-stimulus RSA to stimulus IBI as a function of group accounted for ~49% of the variance in stimulus IBI. Additionally, parameter estimates indicated that accounting for group, pre-stimulus RSA was a predictor for stimulus IBI (*b* = 41.68; *p* < 0.001) such that for each unit increase in pre-stimulus RSA, there was an associated increase of 41.7 ms in IBI. Further, a lack of significant group-by-RSA interaction terms indicated that the effect of pre-stimulus RSA on stimulus IBI did not differ as a function of group (see Table 3).

**Table 3:** Regression model of pre-stimulus RSA to stimulus IBI.

	***b***	**SE(*b*)**	***p***
Intercept	352.40	56.52	<0.001
Pre-stimulus RSA	41.68	8.80	<0.001
FXS + ASD	−69.71	70.88	0.33
FXS-only	42.34	88.65	0.63
nsASD	28.69	69.54	0.68
FXS + ASD x Pre-stimulus RSA	18.74	11.70	0.11
FXS-only x Pre-stimulus RSA	−11.03	14.10	0.44
nsASD x Pre-stimulus RSA	−8.13	11.05	0.46

### Association Between Startle Response and Non-verbal Mental Age, ASD Severity, and Parent-Reported Anxiety (RQ 2)

Lastly, Pearson correlations were tested to assess if non-verbal mental age, ASD severity, or parent-reported anxiety predicted stimulus IBI or post-stimulus IBI within each group. NVMA was significantly correlated with stimulus IBI for the NT group (*r* = 0.63, *p* < 0.001) and FXS-Only group (*r* = 0.50, *p* = 0.022) and for post-stimulus IBI for the NT group (*r* = 0.53, *p* = 0.004) and for the ASD group (*r* = 0.36, *p* = 0.022). The FXS+ASD group did not show an association between NVMA and cardiac response to stimulus (*p* > 0.273). ADOS-2 CSS was moderately correlated with stimulus IBI (*r* = 0.44, *p* = 0.07) and significantly correlated with post-stimulus IBI (*r* = 0.53, *p* = 0.02) for the FXS-Only group, but no relationship was found for any other groups. Lastly, PAS Total Anxiety Raw Score was not significantly correlated with stimulus IBI or post-stimulus IBI for any group (Table 4).

**Table 4:** *Post-hoc* correlations.

		**ADOS-2 CSS**	**NVMA**	**Spence PAS**
nsASD	Startle IBI Post-startle IBI	*r* = −0.13 *r* = −0.10	*r* = 0.26 *r* = 0.36^*^	*r* = 0.22 *r* = 0.23
FXS-only	Startle IBI Post-startle IBI	*r* = 0.44 *r* = 0.53^*^	*r* = 0.50^*^ r = 0.42	*r* = −0.17 *r* = −0.08
FXS + ASD	Startle IBI Post-startle IBI	*r* = −0.19 *r* = −0.28	*r* = 0.12 *r* = 0.29	*r* = −0.07 *r* = −0.02
NT	Startle IBI Post-startle IBI	*r* = 0.27 *r* = 0.22	*r* = 0.63^**^ r = 0.53^**^	*r* = −0.13 *r* = −0.23

* *Significant at p < 0.05;*

** *Significant at p < 0.01*.

## Discussion

Poor physiological regulation in response to threat is linked to a range of negative developmental outcomes including anxiety, behavioral difficulties, and low adaptive skills, which are highly prevalent and impairing in young children with neurodevelopmental disabilities, like FXS and ASD (2–4). In this study, we examined the cardiac response to an auditory startle in an age-matched sample of pre-school children with FXS, ASD, and typical development. We also investigate clinical features that are thought to be associated with poor physiological regulation. Our results indicate that the children with ASD exhibit an exaggerated cardiac response to a sudden auditory stimulus that differentiated them from NT children, but no other significant group differences were observed. Elevated pre-startle RSA was associated with longer IBI at the stimulus across all the groups. In contrast, more severe ASD symptoms were associated with reduced cardiac startle only in the group with FXS that did not have ASD. Also, elevated non-verbal ability was related to reduced cardiac startle in the NT, nsASD, and FXS group without ASD but not in the FXS group with ASD. Finally, parent-reported anxiety symptoms were not associated with cardiac startle in any group.

### Cardiac Auditory Startle Responses Across Groups

The present study found that pre-school children with nsASD showed greater cardiac startle to an auditory startle relative to NT peers. Our findings are consistent with previous studies of individuals with ASD that indicate physiological dysregulation within this population (2, 23, 46, 47). While abnormalities in arousal are consistent in ASD, specific patterns of physiological response to stress are varied, possibly reflecting the heterogeneity of the ASD population. The present study is the first to assess cardiac startle response in young children with ASD and ID, as the majority of previous studies were conducted with individuals with higher cognitive and language abilities or older individuals with ASD. Given the heterogeneity of ASD populations, demonstrating hyperarousal in a relatively homogeneous sample of pre-school children with ASD and ID compared to an age-matched sample shows that this trend toward hyperarousal begins early in life.

Understanding patterns of physiological activity in children with ASD is important, as hyperarousal has been theorized to underlie behavioral and learning difficulties often present in ASD. A review by White et al. (47) posits that physiological hyperarousal in ASD is linked to emotion dysregulation, which can lead to social and psychological difficulties like anxiety. Emotional and behavioral problems can impact early learning and compound existing developmental delays and deficits. For instance, children with FXS and ASD often have more delays early in infancy and behavioral difficulties that cause long-term impairment, including the ability to become independent (48–53). It is essential to understand the presentation of physiological abnormalities in ASD early in life in order to improve developmental outcomes, including social and emotional health (23, 54).

Our findings show interesting parallels with two studies assessing startle responses in individuals with FXS. First, a study by Cohen et al. (45) compared physiological reactivity (electrodermal, heart activity, eye blink) in males aged 10–17 years old with ASD, FXS+ASD, FXS-Only, and NT controls as they viewed emotional stimuli. Similar to the present study, Cohen et al. (45) found that the two FXS groups showed similar patterns of cardiac reactivity, despite the presence of ASD. In contrast, in the Cohen et al. study (45), the FXS groups showed higher cardiac activity than the ASD group, whereas our results identified elevated cardiac activity in the nsASD group compared to the FXS groups. Because the present study consisted of young children, one possible explanation for differences is increased arousal over age in individuals with FXS (2, 29). Further, the present study included females and children with intellectual disabilities, which warrants continued investigation into these factors and the influence they have on cardiac reactivity in individuals with ASD and FXS.

The second study assessed the cardiac response to an auditory startle in boys with FXS aged 1–10.5 years old compared to a NT age-matched group (31). Similar to the present study, the Roberts et al. (31) results did not find significant differences in cardiac response to startle between the boys with FXS and the NT boys. The study did find that as children with FXS aged, their cardiac arousal to the startle increased, a shift not seen in the NT group. Although the developmental shift toward hyper-arousal with age for individuals with FXS was not seen in the present study, the sample was limited to pre-school-aged children and was cross-sectional, rather than longitudinal. Further, the present study examined the cardiac response in a sample of children with FXS divided into those with and without comorbid ASD, which may have implications for the trajectory of arousal in FXS. Future studies should examine the longitudinal patterns of cardiac response to threat in these two groups for nuances in trajectories as children age into adolescence and the potential impacts of comorbid ASD.

### Relationship Between Cardiac Auditory Startle and Clinical Features

In the second aim, we examined whether clinical features of ASD and FXS are associated with cardiac startle responses within and across groups. Cognitive level is particularly important to consider, as autonomic regulation has been linked to aspects of development, like language ability and adaptive functioning, but has not been assessed directly with cardiac startle in young children with intellectual disabilities. Interestingly, NVMA was strongly correlated with post-startle IBI in that, as non-verbal abilities increased, heart rate decreased. The NT group showed a moderate positive relationship between non-verbal ability and startle and post-startle IBI, suggesting that non-verbal abilities and the physiological regulation during an auditory startle are moderately linked in typical pre-schoolers. One hypothesis is that children with higher non-verbal cognitive abilities are able to regulate their cardiac response better than children with lower cognitive abilities because they can interpret the startle as non-threatening. Children with lower cognitive abilities, however, showed difficulty modulating their cardiac responses to the startle, suggesting that cognitive delays negatively impact physiological regulation to threat.

Within the clinical groups, the FXS-Only group also showed positive correlations between non-verbal ability and startle response like the NT group, while the nsASD group and the FXS+ASD group showed no relationship between NVMA and startle response. The shared behavioral diagnosis of ASD might indicate that features of ASD are confounding or influencing the relationship between cognitive ability and startle response. Some studies have found that cardiac flexibility is positively related to cognitive ability in ASD, whereas individuals with ASD and low cognitive abilities show higher physiological arousal and less flexibility in response to threat (23). Overall, the relationship between physiological response to startle and cognitive ability suggests that developmental delays are connected to cardiac startle in that higher non-verbal ability might support the ability to regulate physiologically because of the ability to cognitively cope in response to the startle.

Autism severity also significantly impacts development and the ability to regulate in response to stressors. The link between ASD severity and poor RSA is well-established (1, 55) and the clinical overlap of anxiety symptoms and features of ASD has been observed in neurotypical and ASD populations (55–58). Even with these established connections, no studies have assessed the relationship between cardiac response to startle and ASD severity within ASD and FXS samples. In the present study, the FXS-Only group showed a significant relationship between post-startle heartrate and ASD severity in that the higher the severity score, the less reactive the heartrate. This relationship was specific to the FXS-Only group, which uniquely represents a diagnostic group with ASD symptoms but not ASD. In the FXS-Only group, ASD severity might represent aspects of ASD behaviors like social avoidance or repetitive and restrictive behaviors that suggest a link between specific ASD features and decreased cardiac startle. Interestingly, neither the ASD+FXS group nor the nsASD group showed a relationship between ASD severity and startle response, suggesting that having ASD might overpower any relationship between ASD severity and startle response.

Lastly, a relationship between parent-reported anxiety and cardiac reactivity was not found in any group. The relationship between anxiety and cardiac activity is inconsistent across ASD and FXS, with some evidence suggesting that the interplay between anxiety, ASD severity, and cognitive level makes it difficult to isolate the impact of anxiety alone on autonomic functioning (2, 59). Anxiety in young children, especially those with developmental delays, is very difficult to accurately distinguish from other behavioral difficulties when present (56, 60–62). The reliance on parent reports for interpreting pediatric anxiety is limiting, as many of these measures are designed for neurotypical children with classic presentations of anxiety. Previous research in anxiety in ASD has suggested that higher functioning individuals show higher levels of anxiety, but these studies often rely on classic presentations of anxiety. Individuals with ASD and ID have shown increased problem behavior, elevated heart rate, and decreased RSA in anxiety-provoking situations, which is difficult to characterize as anxiety without multiple sources of data or a functional behavior assessment (62). Thus, to accurately understand and intervene in anxiety in individuals with ID, a multi-method approach that evaluates observed behaviors, physiological data, and clinical interviews is essential (62, 63).

Anxiety often becomes more distinct and easier to identify as a child ages and has a greater ability to use language to report internal feelings and experiences. Thus, anxiety in a pre-school sample with intellectual disabilities is not only challenging to measure, but may be subtle, idiosyncratic, or even absent at this stage of development. Additionally, physiological dysregulation has been posited to underlie emotion dysregulation, and thus, cardiac activity during the startle paradigm might better reflect general emotional dysregulation rather than anxiety specifically (47). Since, autonomic flexibility is associated with social functioning, language ability (3), and behavioral problems (1), the startle paradigm might capture these aspects of development rather than anxiety. In the present study, ASD severity and non-verbal cognitive ability exhibited a correlation to cardiac startle response where parent-reported anxiety did not.

### Limitations

Although this is the first study to directly compare heart activity in pre-school children with ASD and FXS, some limitations should be considered. First, our groups varied in size and variability, which could mask patterns or effects in the smaller groups, particularly the FXS split sample. Additionally, while the PAS has been used in pre-school children with ID, the measure was developed for typically developing children, and thus the nuances of atypical anxiety in children with ASD and FXS (56) might be missed. Further, the PAS is a parent-reported measure, as self-report is very challenging in a young sample with ID. While parent-report is necessary during the assessment of children, it is limited to the parent's perceptions, observations, and conclusions about their child's behavior. The addition of behavioral observations and a clinician-led anxiety interview developed for children with ASD could clarify the relationship between physiological regulation and anxiety in pre-school children with neurodevelopmental disabilities.

## Conclusions and Future Directions

Individuals with neurodevelopmental disabilities often show difficulties with autonomic flexibility in response to aversive stimuli and experience emotional and behavioral dysregulation alongside physiological dysregulation (2, 64). The present study assessed cardiac startle response in children with varying risks for anxiety, ASD, and cognitive delays. The results demonstrate that physiological dysregulation begins early in childhood, during windows of time when children are particularly sensitive to intervention (65–67). Children with autonomic flexibility have better language, cognitive, and social skills (3, 23), and thus, the relationship between behavioral and physiological functioning must be considered in early interventions. Evidence suggests that incorporating physiological components, like relaxation and neurofeedback, with learning social and cognitive skills can lead to more skill acquisition by individuals with ASD (68–71). Therefore, interventions addressing physiological regulation in response to environmental stressors paired with skill-building components can prime the child to be more receptive to interventions targeting sleep, attention, and learning.

In order to ascertain the nuances of physiological regulation and behavioral problems in young children with neurodevelopmental disabilities, future work should incorporate behavioral observations alongside physiological measures. Additionally, integrating measures of sensory behaviors and emotion regulation could elicit insight into the interplay of behavioral, physiological, and neurological factors impacting child development. Further, studies in older and higher-functioning samples of ASD have found differences in physiological response when groups are divided into subgroups of high and low anxiety (27, 72). Thus, distinguishing clinical groups by anxiety symptoms might delineate the relationship between high anxiety symptoms and physiological reactivity. Lastly, including clinical interviews of anxiety that target both traditional and atypical presentations in young children are important to clarify the role of anxiety in high-risk populations. Overall, a richer understanding of the complex relationship of physiological markers and behavior difficulties in young children with neurodevelopmental disabilities is a critical step in developing and refining appropriate interventions for early childhood.

## Data Availability Statement

The raw data supporting the conclusions of this article will be made available by the authors, without undue reservation.

## Ethics Statement

The studies involving human participants were reviewed and approved by University of South Carolina Institutional Review Board. Written informed consent to participate in this study was provided by the participants' legal guardian/next of kin.

## Author Contributions

JR served as principal investigator of the parent study. JE and JR conceptualized the paper and finalized the paper. JE, JR, and AH drafted the manuscript. EW consulted on the analytic plan and reviewed the section Results. KS was instrumental in the collection and processing of cardiac recordings. All authors provided feedback to early drafts.

## Funding

This study was supported by NICHD: F32HD097877, PI: EW and NIMH: R01MH090194-06 and R01MH107573-01A1, PI: JR.

## Conflict of Interest

The authors declare that the research was conducted in the absence of any commercial or financial relationships that could be construed as a potential conflict of interest.

## Publisher's Note

All claims expressed in this article are solely those of the authors and do not necessarily represent those of their affiliated organizations, or those of the publisher, the editors and the reviewers. Any product that may be evaluated in this article, or claim that may be made by its manufacturer, is not guaranteed or endorsed by the publisher.
